# Collagen and Microvascularization in Placentas From Young and Older Mares

**DOI:** 10.3389/fvets.2021.772658

**Published:** 2022-01-04

**Authors:** Ana Catarina Neto da Silva, Ana Luísa Costa, Ana Teixeira, Joana Alpoim-Moreira, Carina Fernandes, Maria João Fradinho, Maria Rosa Rebordão, Elisabete Silva, José Ferreira da Silva, Miguel Bliebernicht, Graça Alexandre-Pires, Graça Ferreira-Dias

**Affiliations:** ^1^Faculdade de Medicina Veterinária, CIISA - Centro de Investigação Interdisciplinar em Sanidade Animal, Universidade de Lisboa, Lisboa, Portugal; ^2^Embriovet, Muge, Portugal; ^3^Pole Reprodución Haras de La Gesse, Boulogne-sur-Gesse, France; ^4^Coimbra College of Agriculture, Polytechnic Institute of Coimbra, Coimbra, Portugal

**Keywords:** collagen, fibrosis, placenta, age, mare, foal, microvascularization, gestation

## Abstract

In older mares, increasing collagen fibers (fibrosis) in the endometrium and oviduct predisposes to sub-fertility and infertility. In this study, (i) gene transcription of collagen (qPCR: *COL1A1, COL1A2, COL3A1, COL5A1*); (ii) total collagen protein (hydroxyproline); (iii) collagen distribution (Picrosirius red staining; polarized light microscopy); and (iv) microvascular density (Periodic acid-Schiff staining), were evaluated in mares' placenta, and related to mares age, and placenta and neonate weights. Samples were collected from the gravid horn, non-gravid horn, and body of the placenta from younger (*n* = 7), and older mares (*n* = 9) of different breeds. Transcripts of *COL1A1, COL3A1* and *COL5A1*, total collagen protein, chorionic plate connective tissue thickness, and microvascularization increased in the gravid horn of older mares' placentas, compared to the youngest (*P* < 0.05). Although in other species placenta fibrosis may indicate placental insufficiency and reduced neonate weight, this was not observed here. It appears that older fertile mares, with more parities, may develop a heavier, more vascularized functional placenta with more collagen, throughout a longer gestation, which enables the delivery of heavier foals. Thus, these features might represent morphological and physiological adaptations of older fertile mares' placentas to provide the appropriate nutrition to the equine fetus.

## Introduction

The placenta is an overly complex organ of vital importance in equine pregnancy. Mare's placenta as epitheliocorial type, enables the direct contact of the endometrial epithelium with the chorionic surface, through six layers of tissue separating the maternal circulation from the fetal circulation, throughout the entire pregnancy (1–3). This organ allows the fetus to be nourished through metabolic exchange of nutrients, oxygen supply and elimination of debris, providing protection against internal and external aggressions (4). As a microcotyledonary and diffuse placenta, it also presents a uniform distribution of chorionic villi on the maternal surface, which attach to the maternal epithelium forming well distinguished microcotyledons (2, 3).

In equine clinical practice, the meticulous exam of the placenta, mainly by gross anatomy inspection, is a routine procedure (5). In the mare, placenta alterations can be a sign of malfunction. Despite the proposed diagnostic blood markers, the value of an experiencing clinician examining the mare is essential (6). It is well known that intrauterine fetal nutrition influences both foal's health (7, 8), and its athletic performance (9, 10). Nevertheless, regardless of the animal species, the macroscopic examination of the placenta does not reveal microscopic lesions that are only detected by histological examination or electron microscopy observation, and that might be related to fetal death, neonate low body weight or sickness (11–14).

Aging of the mares has been related to increased fibrosis in the endometrium (15, 16), and oviduct (17), and several pathological alterations in the placenta (13, 18). In fact, during mare gestation, besides morphological alterations, and degenerative changes in the microplacentomes on the surface of the mare placenta, also the development of fetal and maternal capillary beds within each microplacentome is diminished, disturbing physical and hematological contact at the fetomaternal interface, and hence impairing fetal growth (13, 18). In other species, vascularization of the placenta is also extremely important, as in the sow (11), bitch (14) and in woman (19). In fact, in humans, reduced vasculature of the placenta is frequently related with intrauterine fetal growth restriction (19).

The fibrotic tissue is characterized by the excessive deposition of extracellular matrix components, such as collagen, fibronectin and hyaluronic acid, due to the activation, proliferation and accumulation of fibroblasts and myofibroblasts (20). In this manner, fibrotic tissue can deregulate the normal functioning and architecture of an organ (21). Fibrosis develops from a chronic inflammatory process, in which both the mechanisms of innate and acquired immunity play an important role (22), and where inflammation and tissue remodeling occur simultaneously (23). Fibroblasts, once activated, differentiate into myofibroblasts which initiate the deposition of connective tissue components, remodeling and progressively destroy the tissue architecture (24). Even though the collagen existing in human placenta is related to the normal development of the placenta, it has also been associated to metabolic complications of placental function (25). Collagen types I, III, IV, and V are actively involved in the formation of the fibrotic process of human placenta's chorionic villi (26).

A body of evidence shows that in a number of species, deficiencies in placental structure and function can be reflected in impaired fetal development and growth with the decrease in neonates birth weight (13, 14, 18, 27). Therefore, we have put forward the hypothesis that as mares age, fibrosis increases in the placenta, and microvascularization decreases, leading to a reduction in foal weight. Thus, the objective of this study was to evaluate in three portions (gravid horn, non-gravid horn and body) of mares' placenta (i) gene transcription of collagen (*COL1A1, COL1A2, COL3A1, COL5A1*); (ii) total collagen protein (hydroxyproline); (iii) collagen distribution in histological sections; and (iv) microvascular density, and relate them to mares age, and placenta and neonate birth weights.

## Materials and Methods

### Mare's Identification

Sixteen pregnant mares of different breeds (Lusitano, French Trotter, Anglo-Arabian, Hanoverian and KWPN) from the same stud-farm were randomly selected and grouped into two categories, according to their age. The youngest mares, with ages between 5 and 9 years old, were allocated to the “Young mares” group (*n* = 7; 6.4 ± 0.6 years, mean± SEM), while the oldest mares, aged 10–15 years old, were included in the “Older mares” group (*n* = 9; 12.2 ± 0.5 years). They foaled between April and May.

The mares were kept on pasture and diets were adjusted according to different needs. All the mares were routinely dewormed and vaccinated according to stud-farm protocol. In the last month of gestation mares were individually stabled overnight, for supervision of the deliveries. At foaling, the placentas of those mares were immediately collected after expulsion, weighed, and used for sampling. New-born foals were weighed within 24 h after delivery.

### Sample Collection

**Figure 1:**
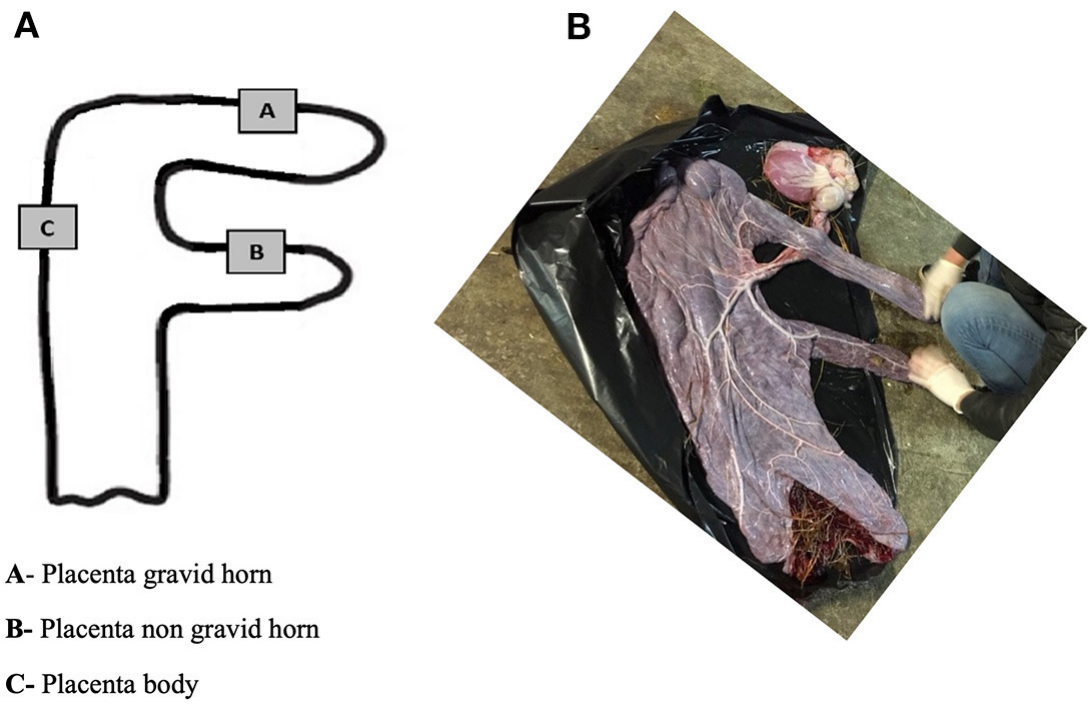
Diagrammatical representation of the placenta sites for sample collection **(A)**. Photo of the allantoic surface of a mare's placenta after delivery **(B)**.

Different regions of the placenta were identified as gravid-horn (A), non-gravid horn (B) and placental body (C) (Figure 1). From each of these areas, tissue samples of approximately 5 mm3 were collected, as follows: (i) each one of two pieces was immersed in 1 mL of RNA Later® (AM7020, Ambion, Applied Biosystems, Waltham, Massachusetts, USA) for real-time PCR (qPCR) studies, and for determination of total collagen by immunoassay; and the other two samples were placed in 4% buffered formaldehyde for histological analysis (microvascularization assessment and collagen fibers distribution). Placenta samples in RNA Later® were kept at 4°C for 24 h and then stored at −80°C until laboratory tests were carried out.

### Quantitative Real-Time Polymerase Chain Reaction

Determination of the gene transcription of collagen genes, such as type I (*COL1A1, COL1A2*), type III (*COL3A1)* and V *(COL5A1*) was performed by real-time PCR (qPCR) in the gravid horn, non-gravid horn, and body of the placenta obtained from younger mares (*n* = 7); and older mares (*n* = 9). Specific primers were designed, as well as the reference gene, using the Internet based program Primer-3 and Primer Premier software (Premier Biosoft Interpairs, Palo Alto, CA, USA). The primers used are listed in Table 1. Extraction of RNA from placenta samples was accomplished with the alcohol method. Briefly, placental tissues (50mg) were macerated with a scalpel blade. Then, 500 μL (10 times the sample weight, in mg) of TRI® reagent (T9424; Sigma Life Science, Burlington, MA, United States) were added, and sample disruption was performed with the TissueLyser II (Qiagen, Hilden, Germany) for five cycles of 30 s each, at 25 Hz, and centrifuged at 12,000 g for 10 min at 4°C. For RNA separation, 100 μL (0.2 of the supernatant volume) of chloroform (C2432-1L – Sigma-Aldrich, Saint Louis, Missouri, USA) were added to the collected supernatant. The solution was mixed, incubated on ice for 5 min, and then centrifuged at 12,000 g for 20 min at 4°C. The supernatant was collected. Afterwards, 250 μL of isopropanol (Isopropyl Alcohol - 0918 – 500 ML - Biotechnology- VWR Life Science; Radnor, Pennsylvania, USA), were added to 500 μL of the supernatant, incubated for 10 min, and centrifuged at 12,000 g for 20 min at 4°C, to precipitate the RNA. The recovered pellet was washed with 75% ethanol (K43342083215, Index -No: 603-002-00-5, Merck KGaA, Darmstadt, Germany; volume equal to the supernatant volume), centrifuged at 7,500 g for 5 min, at 4°C, and air-dried at room temperature for 30 min. RNA was dissolved into 20–30 μL DEPC-treated water (AM9915G; Thermo Fisher Scientific, Waltham, Massachusetts, USA), and stored at −80°C. RNA concentration and quality were evaluated using Nanodrop® (ND200C; Thermo Fisher Scientific, Waltham, Massachusetts, USA), and by visualization of rRNA bands after electrophoresis in a 1.5% agarose gel and red staining (41003; Biotium, Hayward, CA, USA). The extracted RNA was used for cDNA synthesis, through reverse transcription. The latter was performed using M-MLV Reverse Transcriptase (M170B, Promega®, Madison, Wisconsin, USA) from 1 μg total RNA in a 20 μL reaction volume using oligonucleotides (C1101, Promega®, Madison, Wisconsin, USA) and an RNase inhibitor.

**Table 1:** Primer sequences used in real time PCR analysis of mare.

**Gene (access number)**	**Sequence 5^**′**^ – 3**	**Amplicon base pairs**
*COL1A1* (100033877)	Forward: TATGGAAACCCGAGCCCTG Reverse: ACTCCTGTGGTTTGGTCGTCTG	175
*COL1A2* (XM_001492939.3)	Forward: CAAGGGCATTAGGGGACACA Reverse: ACCCACACTTCCATCGCTTC	196
*COL3A1* (AF117954.1)	Forward: CAAAGGAGAGCCAGGAGCAC Reverse: CTCCAGGCGAACCATCTTTG	98
*COL5A1* (100069057)	Forward: CGCTCTCCCGTCTTCCTCT Reverse: TGCCGAACACGATGATGC	228
*GAPDH* (NM_001163856.1)	Forward: CACCCACTCTTCCACCTTCG Reverse: CTTGCTGGGTGATTGGTGGT	173
*RPL32* (XM_001492042.6)	Forward: AGCCATCTACTCGGCGTCA Reverse: GTCAATGCCTCTGGGTTTCC	144
*β2M* (X69083)	Forward: CGGGCTACTCTCCCTGACTG Reverse: TTGGCTTTCCATTCTCTGCTG	92

*COL1A1, collagen, type I, alpha 1; COL1A2, collagen, type I, alpha 2; COL3A1, collagen, type III, alpha 1; COL5A1, collagen, type V, alpha 1; GAPDH, glyceraldehyde 3-phosphate; RPL32, ribosomal protein L32; β2M, beta-2-microglobulin*.

The obtained cDNA was combined with Power SYBER Green PCR Master Mix (Ref. 4368706; Applied Biosystems, Waltham, Massachusetts, USA) and with the target or reference genes, and qPCR assays of both were performed simultaneously in a StepOne-Plus TM Real-Time PCR System (Applied Biosystems, Waltham, Massachusetts, USA), using the universal temperature cycles previously described (28).

Before running the assay, primers concentration was optimized (80 nM for target and reference genes), and the reference gene was validated. To determine the most stable internal control gene, four potential reference genes were initially considered, as follows: glyceraldehyde 3-phosphate dehydrogenase (*GAPDH*), succinate dehydrogenase A complex, subunit A, flavoprotein (*SDHA*), beta-2-microglobulin (β*2M*) and ribosomal protein L32 (*RPL32*). *GAPDH* was the most stable internal control gene (29), and therefore considered our reference gene.

Confirmation of the specificity of the PCR products was obtained by running a 2.5% agarose gel (BIO-41025; Bioline, Luckenwalde, Germany) and by the analysis of the melting curve. Quantitative analysis of mRNA was determine by real-time PCR Miner algorithm (30), as previously described (31). Levels of mRNA of the target genes were normalized against those of the reference gene.

### Determination of Total Collagen Concentration

As the transcription of genes does not always correspond to the amount of protein synthesized (32), there was the need to quantify the total collagen present in the placenta samples. Since hydroxyproline is a major component of collagen that stabilizes its helical structure, determination of hydroxyproline concentration can be used as an indicator of collagen presence (33). Thus, total hydroxyproline concentration in the various portions of all the equine placentas was assessed by using an enzyme immunoassay method (Ref. ABIN593448; Hydroxyproline colorimetric assay kit, Antibodies-online GmbH, Aachen, Germany). A colorimetric product proportional to the hydroxyproline present in the sample was obtained and subsequently read on a spectrophotometer at the wavelength of 560 nm, according to the manufacturer's instructions. Results were expressed as micrograms of hydroxyproline per milligram of placenta.

### Qualitative Histological Evaluation of Collagen

Placenta samples preserved in 4% formaldehyde were dehydrated in increasing concentrations of ethanol (70, 80, 95, and 100%), and in xylol, and then processed to obtain paraffin blocks. Histological sections (4 μm thick) of each chosen site of the placenta (gravid horn, non-gravid horn, body) were stained with Picrosirius Red (PSR). The evaluation of the PSR stained samples was performed using a camera (TIS 2MP RGB) coupled to a vertical wide-field microscope (Olympus BX51, Olympus Corporation, Tokyo, Japan) in a 100x magnification, under a polarized light beam. From each slide stained with PSR, 10 images of each portion (A, B, and C) observed in a dark field were randomly obtained. In addition, to identify the structures photographed, duplicates of those photographs taken under polarized light were also taken in bright field. Since PSR stain is particularly useful to depict the organization and/or collagen fiber orientation in various connective tissues under normal or pathological conditions (34), this method was used to assess collagen in mare's placenta.

### Measurement of the Chorionic Plate Connective Tissue Thickness

The thickness of the chorionic plate connective tissue in the gravid horn of mares' placentas was evaluated on 4 μm histological sections, stained with hematoxylin-eosin (05-06014E; Bio-Optica; eosin HT1103128; Sigma-Aldrich, Saint Louis, Missouri, USA), observed under a light microscope (Leica DM500), and photographed at 100x magnification. Two photographs of the chorionic plate of the gravid horns of each placenta were randomly selected. Later, 10 measurements were made from each photograph using the program *ImageJ* (National Institute of Health, USA), which is an open source software for processing and analyzing scientific images. Once the 20 measurements from each placenta were gathered, they were subjected to statistical analysis.

### Microvascular Density

From each portion of the placenta (gravid horn, non-gravid horn, and body) preserved in paraffin blocks, 4 μ-thick histological tissue sections were retrieved, and stained with Periodic Acid Shiff reagent (PAS; VWR Chemicals, ref. 20593.151, Leuven, Belgium). This stain has been largely used as a marker of endothelial cells, since it strongly reacts with carbohydrates present in the microvascular basement membrane (35). In this study, blood vessel walls were considered equally regardless of their nature (arterioles, venules, or capillaries) (28). The histological slides were observed under light microscopy and photographed at a total magnification of 100x, for further imageJ program analysis. Number of vessels and microvascular areas were determined on 10 randomly chosen microscope fields of each portion of the placentas. The percentage of the area occupied by blood vessels, with respect to the entire area of the micrograph, was considered as the vascular area and calculated for all 10 microscopic fields, from each portion of each placenta. On the same micrograph areas, the number of vessels present were also counted. Mean values of total vascular area and total blood vessel number for each placenta region were further considered for statistical analysis (28, 36).

### Statistical Analysis

Statistical analysis was performed using the *GraphPad Prism* (version 8.4.3), *Statistica 7* and *SAS* (SAS 9.4 Institute Inc., Cary, NC, USA) programs. On a first approach and after checking the normal distribution of the variables with Shapiro-Wilk test, *COL1A1, COL1A2, COL3A1* and *COL5A1* mRNA transcription, hydroxyproline quantification and chorionic plate thickness were analyzed by one-way ANOVA, followed by Fisher's Least Significance Difference (LSD) test. To evaluate the relationship between newborn foal's weight and placenta's weight or mare's age, Pearson's correlation coefficients were calculated, followed by linear regression analysis. In order to evaluate the effects of age (young *vs*. older mare groups), location of placental sampling (A, B, and C) and their interaction on variables, a mixed model was used (MIXED procedure of SAS). The gestation length and parity were used in this analysis as covariate, and the sex of the foal was also tested. When significant differences were detected, the differences among means were evaluated using the Tukey-Kramer test. The level of significance was defined as *p* < 0.05. Data are shown as mean ± SEM.

## Results

### Animals' Data

**Figure 2:**
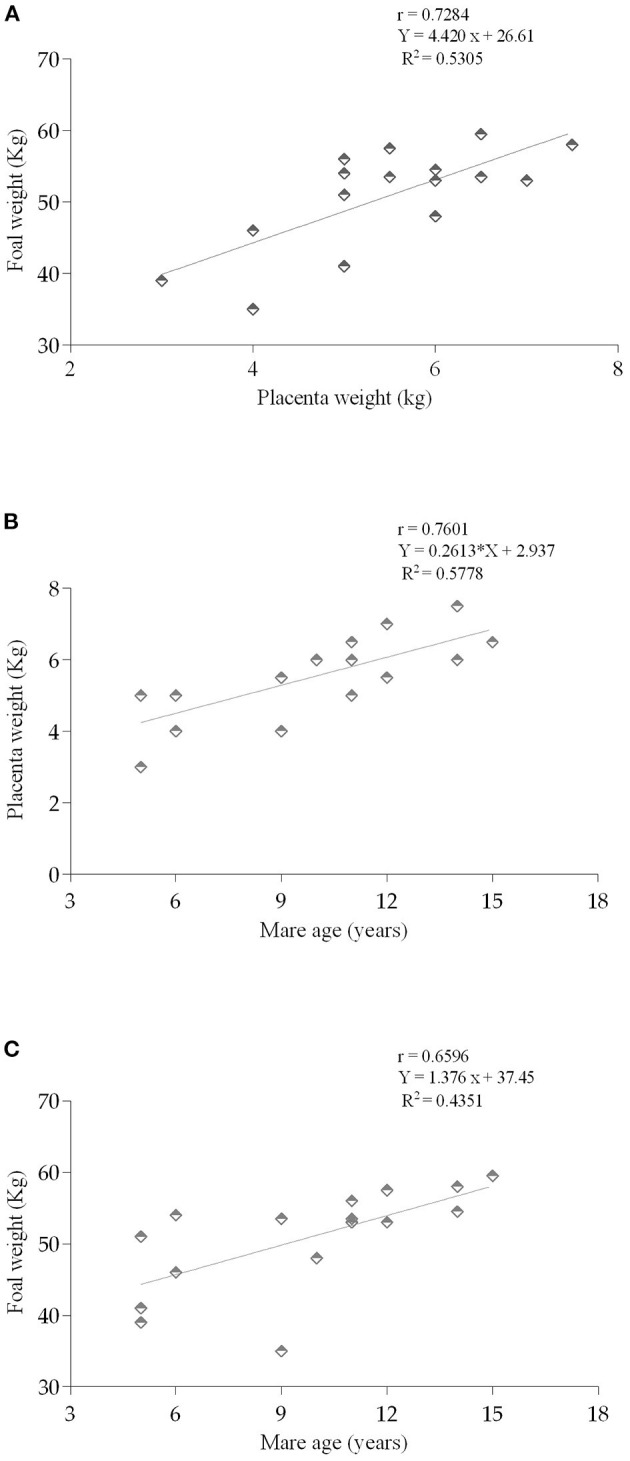
Relationship between the **(A)** weight of the placenta (kg) and foal weight at 24h (kg) (*p* = 0.0014); **(B)** mares age (years) and placenta weight (*p* = 0.0006); and **(C)** mares age and the weight of the foal (kg) (*p* = 0.0054). Pearson correlation (r). Linear regression (R^2^).

The length of the pregnancies that resulted in the foalings from which placentas were retrieved was from 324 days (minimum) up to 352 days (337.2 ± 2.3 days). Placenta weights varied between 3 and 7.5 kg (5.5 ± 0.3 kg), and it increased with mares aging (*p* < 0.001). The weight of the foal at 24 h after foaling ranged from 35 to 59.5 kg (50.8 ± 1.8 kg). Heavier foals were born from older mares (*p* < 0.0001), that also had more parities (*p* < 0.0001), and longer gestations (*p* < 0.01). When considering the mares, according to their age group, data are presented in Table 2. There was a positive correlation between the weight of the placenta and the weight of the foal (r = 0.728; *p* = 0.001; Figure 2A); between the age of the mare and the weight of the placenta (r = 0.760; *p* = 0.0006; Figure 2B); and the age of the mare and the weight of the foal (r = 0.659; *p* = 0.005; Figure 2C). Evaluation of the ratio placenta weight/foal weight showed a higher ratio in older mares when compared to the younger mares (0.114 ± 0.003 vs. 0.099 ± 0.003; *p* < 0.001).

**Table 2:** Age, parity, gestation length, placenta weight (kg), and foal weight (kg) at 24 h post-foaling, in the two groups of mares (mean ± SEM).

	**Age (years)**	**Parity (#)**	**Gestation (days)**	**Placenta weight (kg)**	**Foal weight (kg)**
Young mares	6.4 ± 0.6	1.7 ± 0.2^***^	333.4 ± 1.8^**^	4.5 ± 0.17^***^	45.6 ± 1.2^***^
Older mares	12.2 ± 0.5	4.4 ± 0.2^***^	340.1 ± 1.6^**^	6.2 ± 0.15^***^	54.7 ± 1.0^***^

*Asterisks indicate significant differences between parity, gestation length and placenta weight in young and older mares, as well as between the weight of their foals (*

** *p <0.01*;

*** *p <0.0001)*.

### Quantitative Real-Time Polymerase Chain Reaction

**Figure 3:**
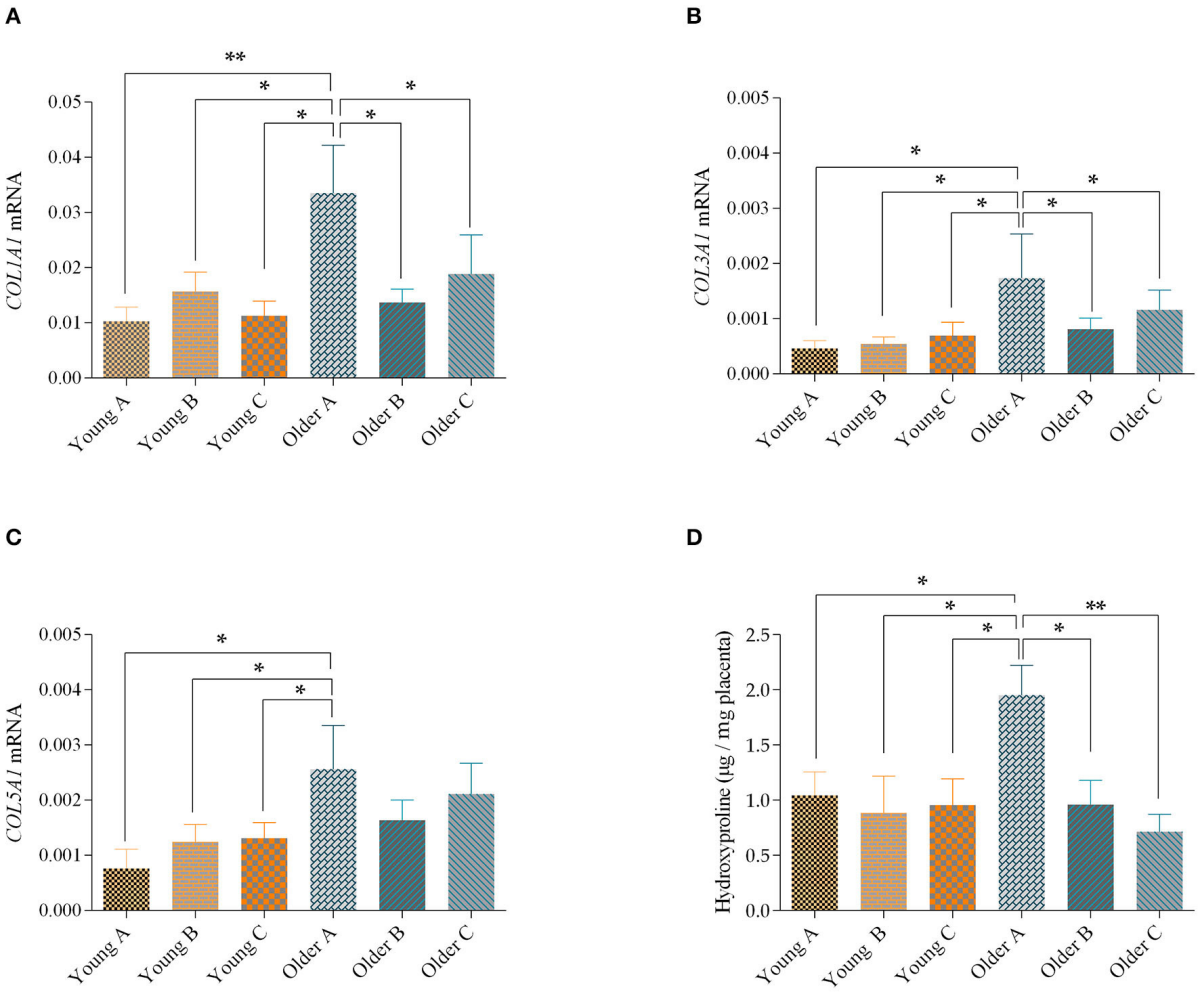
Transcript levels of *COL1A1* **(A)**, *COL3A1* **(B)** and *COL5A1* **(C)** and levels of hydroxyproline **(D)** in the gravid horn **(A)**, non-gravid horn **(B)** and body **(C)** of placentas from young and older mares. Bars represent the mean ± SEM. Asterisks indicate significant differences between the placental portions (*p < 0.05; **p < 0.01). Data were analyzed by one-way ANOVA, followed by Fisher's LSD test.

In older mares' placenta, when comparison was made with the same placenta regions of younger mares, *COL1A1* mRNA transcript levels were higher in the gravid horn (portion A) (*p* = 0.01), and between the non-gravid horn (portion B), or between the placenta body (portion C) (*p* < 0.05). In addition, *COL1A1* transcription in the gravid horn of older mares was also increased, when compared with the non-gravid horn and body of the same placentas (*p* < 0.05; Figure 3A). When gestation length and parity were used as covariates, no significant effects were observed.

Although, no differences were found in *COL1A2* mRNA levels for direct comparisons between the placenta portions of young and older mares, a significant effect of age was observed, with higher levels of *COL1A2* transcripts in older mares (*p* < 0.05). When we used the gestation length and parity as covariates no significant effects were observed.

Regarding *COL3A1* mRNA levels, which were similar to *COL1A1*, increased in the gravid horn of the placentas of older mares, when compared to the same portion in younger mares (*p* < 0.05). Besides, the gravid horn of placentas of older mares had the highest *COL3A1* transcript level, compared to portions B and C of the same placenta (*p* < 0.05; Figure 3B). When gestation length was used as a covariate there was a significant effect of age, with older mares showing higher levels of *COL3A1* mRNA in their placentas than younger mares (*p* < 0.01).

In addition, when the *COL5A1* transcripts levels were assessed in the different portions of the placenta, an increase in mRNA levels was observed in the gravid horn of older mares, compared to any portion of the placenta from younger mares, (*p* < 0.05; Figure 3C). There was a significant effect of age (Young mares *vs*. Older mares) over this parameter (*p* < 0.05). Also, for this parameter, no significant effects were observed when the length of gestation and parity were used as covariates.

Foal sex had no effects on *COL1A1, COL1A2, COL3A1* or *COL5A1* transcript levels.

### Hydroxyproline Concentration

In agreement with the increase in *COL1A1, COL3A1* and *COL5A1* transcripts obtained by qPCR, there was a raise in the hydroxyproline amino acid in the pregnant horn of older mares, relatively to the non-gravid horn (*p* < 0.05), and to the placental body (*p* < 0.01) of the same placentas and to all studied placental portions of the youngest mares (*p* < 0.05; Figure 3D). In addition, hydroxyproline levels in the placenta were not influenced by gestation length or sex of the foal.

### Qualitative Histological Assessment of Collagen in Mare's Placenta

**Figure 4:**
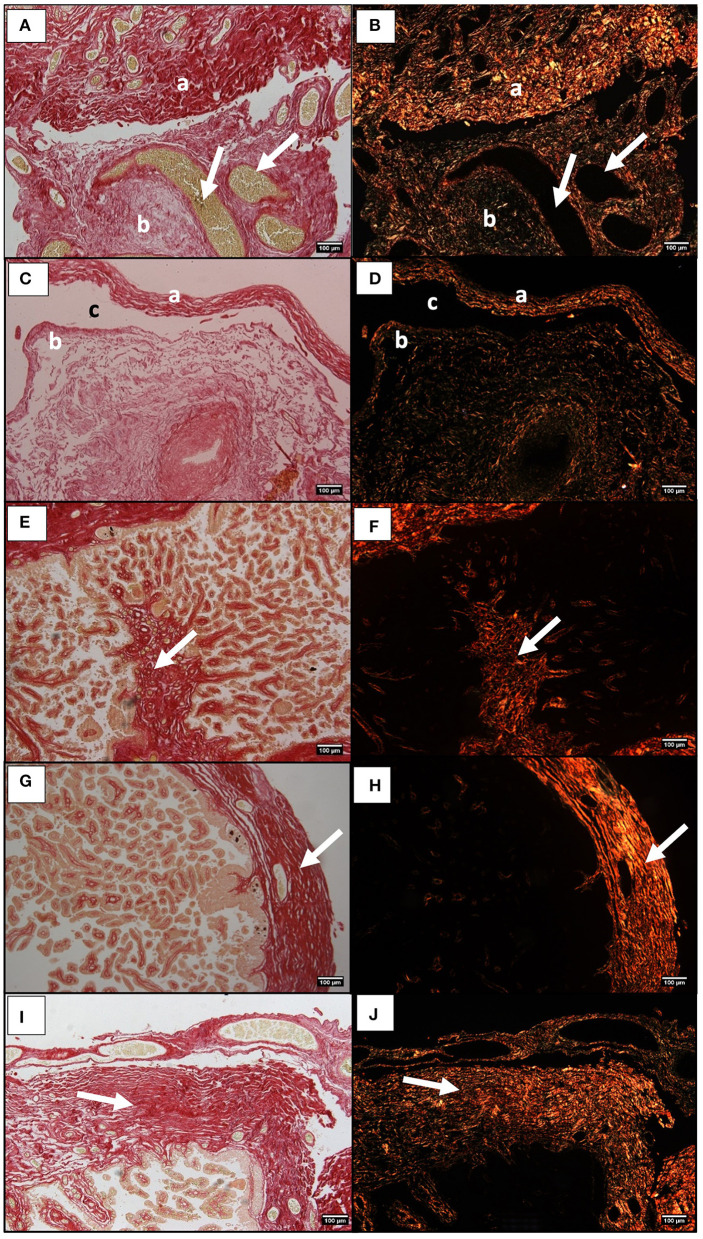
Histological sections of the equine placenta pregnant horn stained with Picrosirius Red and visualized in bright field **(A,C,E)** and under polarized light **(B,D,F)**. Red and green colors reflect the orientation of the collagen bundles. **(A,B)** Chorionic plate connective tissue (a), and allantoic membrane connective tissue (b). White arrows indicate blood vessels. **(C,D)** Connective tissue adherent to the allantoic epithelium (a), and in the external part of the chorionic membrane (b), next to the extracelomic space (c). **(E,F)** collagen fibers in the connective tissue of villi (white arrow). **(G,H)** Chorionic plate connective tissue in the pregnant horn, of a placenta from a young mare (white arrow). **(I,J)** Chorionic plate connective tissue in the pregnant horn, of a placenta from an older mare (white arrow).

The histological observation of mare's placenta, under a bright field and under a polarized light beam showed the presence of collagen mostly in the chrorionic plate and allantoic connective tissue. The qualitative assessment suggests that a greater abundance of fibers might be present in the connective tissue of the chorionic plate of the gravid horn in all mares (Figures 4A,B). As for the allantoic connective tissue, collagen fibers seem to also predominate in large amounts (Figures 4A,B). Whenever the exocelomic space was present, collagen fibers surrounded it, within the inner thin layer of connective tissue adherent to the epithelium of the allantoic surface, more internally (Figures 4C,D), and in the allantoic connective tissue associated to the chorionic plate, externally (Figures 4C,D). Very few collagen fibers were present in the connective tissue of the axis of villi, even though in the gravid horn they appeared to be more abundant (Figures 4E,F). The qualitative histological observation of mares' placentas suggested more collagen was present in the gravid horn, with respect to the non-gravid horn and placenta body, regardless of mares age.

### Chorionic Plate Connective Tissue Thickness

The qualitative observation of collagen fibers stained by PSR in mares' placentas did not reveal any evident change between young and older mares' placentas that could explain the observed increase in *COL1A1* and *COL3A1* mRNA, evidenced by qPCR, and by hydroxyproline in the gravid horn of older mares. Thus, there was the need to measure the thickness of the connective tissue of the chorionic plate in the gravid horn of all placentas. Our results indicate a significant increase in thickness of the connective tissue of the chorionic plate in the gravid horn of older mares (449.1 ± 42.1 μm), when compared to the same structure in the placentas of the youngest mares (401 ± 47.7 μm; *p* < 0.05), as can be visually depicted in Figures 4G–J.

In addition, gestation length was positively correlated with the chorionic plate thickness (r = 0.525; *p* < 0.05) meaning that longer gestations increased the chorionic plate thickness. Mares that foaled a male foal tended to have a thicker chorionic plate (*p* = 0.08).

### Quantitative Assessment of Microvascular Density

**Figure 5:**
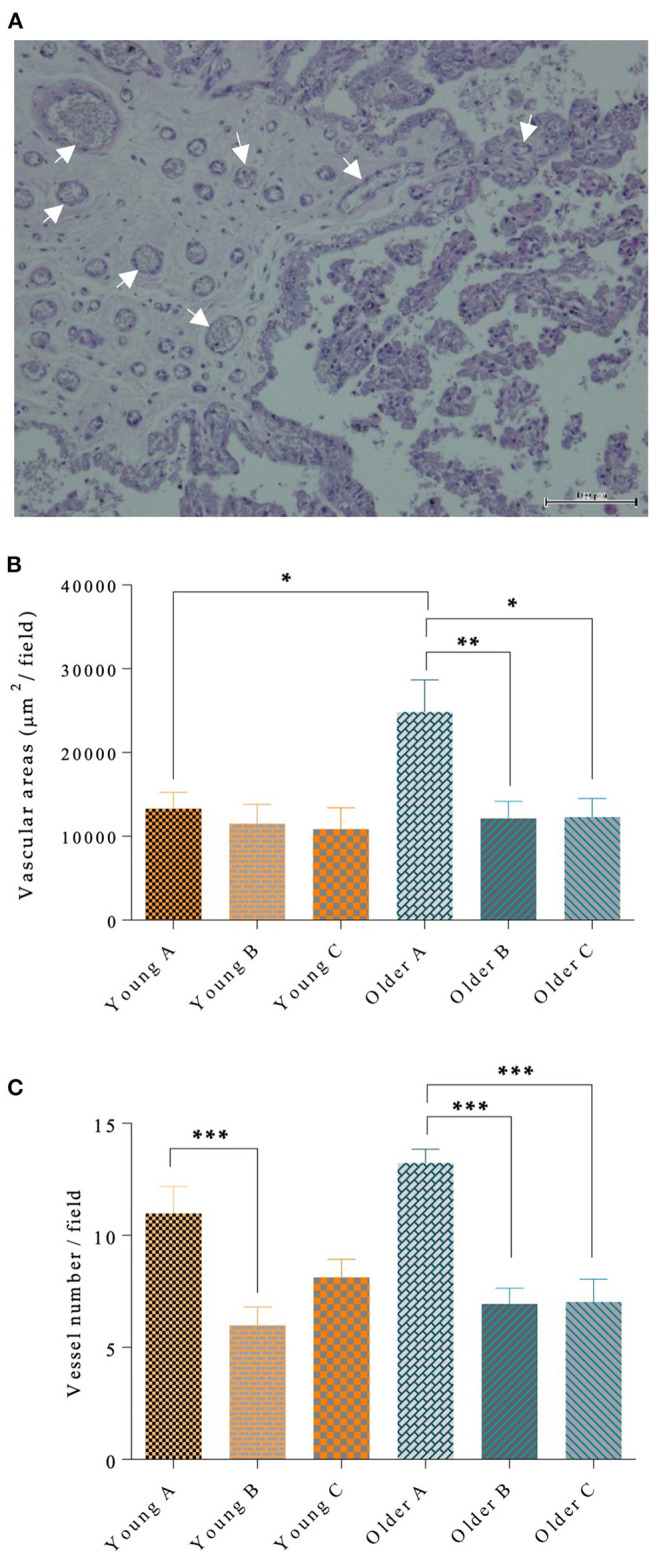
Blood vessel walls stained by PAS (some marked with arrows), in mare placenta **(A)**. In **(A)**, bar = 100 μm. In the left half of the image, the chorion is depicted, with the chorionic plate enclosing vessels, and the trophoblast (simple cuboidal epithelium whose cells possess a basophilic cytoplasm) lines the chorionic plate. In the right half, the chorionic villi can be seen, externally covered by trophoblasts and with the axis composed by connective tissue. In larger diameter villi, fine vessels are observed. Vascular area per μm^2^ **(B)** and vessel number per field **(C)** are shown in the different portions of the placenta in the pregnant horn **(A)**, non-pregnant horn **(B)** and body **(C)** of placentas from young and older mares. In **(B)** and **(C)**, bars represent the mean ± SEM. Data were analyzed by one-way ANOVA, followed by Fisher's LSD test. Asterisks indicate significant differences between the placental portions (*p < 0.05; **p < 0.01; ***p < 0.001).

Blood vessel walls were stained by periodic-Acid shift, as shown in Figure 5A. The microvascular area in the gravid horn of the placenta was larger in older mares than in younger mares (*p* < 0.05; Figure 5B). In addition, in older mares, the vascular area of the gravid horn was increased with respect to the non-gravid horn (*p* < 0.01), and placenta body (*p* < 0.05; Figure 5B). Nevertheless, the vascular area in young mares' placenta was identical in all the evaluated portions of this organ (Figure 5B). Besides the observed increase in the vascular area in the gravid horn of older mares' placentas, the number of vessels per microscopic field was also higher in that region, when compared to the other portions of the placenta (*p* < 0.001; Figure 5C). Older mares' placentas presented larger vascular areas than younger mares' placentas (*p* < 0.05). Gestation length and sex of the foal had no influence on vascular area. However, there was an effect of parity in the vascular area of the gravid horn (*p* < 0.05) with mares that had a higher number of parturitions presenting an increased vascular area.

In younger mares there was an increase in vessel count in the pregnant horn, when compared to the non-pregnant horn (*p* < 0.001; Figure 5C). Gestation length and sex of the foal had no influence on vessel count. But, similarly with the results obtained for vascular area, parity had an effect on the number of blood vessels of the gravid horn (*p* < 0.0001), regardless of the age of the mare.

## Discussion

Earlier studies have mainly dealt with mare placenta histology and cell proliferation patterns during development (37–39). Even though structural and hemovascular aspects of placental growth throughout gestation in young and aged mares have been described (13), collagen content at the histological and molecular levels has not been assessed. Thus, to the best of our knowledge, this is the first study quantifying collagen fibers and microvascularization in the equine placenta, relating them to the different portions of the placenta, mare's age, placenta, and foals' s weight.

In the present study, heavier foals at birth were born from older mares, with more parities, that also delivered heavier placentas, and had longer gestation lengths. There was a positive correlation between foal's weight and placenta's weight, as well as between foal's weight and mare's age. The positive correlation between the total weight of fetal membranes and the weight of the foal at birth observed in the present work, has been shown before for Thoroughbreds (40), and Lusitano mares (41). In fact, in Thoroughbreds, for each kg of increase in placental weight, there was a raise of 4.5 kg in foal's weight (40). In the mare, the weight of the placenta progressively increases with parity, maternal size, and directly affects the placenta transport surface area and function, as well as fetal endocrine glands that mediate fetal development toward term (42–45). In addition, equine placental weight increases in the months of April and May deliveries, in comparison to earlier foalings in the year (46). Since all deliveries of the mares in the present study occurred in April and May, changes in placenta weights may be related to mares age, parities, gestation length or foals' weight, but not to any seasonal effect.

The transcript levels of mRNA showed an increase in *COL1A1, COL3A1* and *COL5A1* in the pregnant horn of older mares' placenta. This raise in collagen transcription was confirmed by hydroxyproline content (total collagen) in the placenta tissues. In fact, post-transcriptional, post-translational and degradation regulation contribute to the concentration of proteins in the tissue (32). In human placenta, collagen changes its composition during placenta development and aging, as pregnancy progresses, for providing nutrients to the fetus and maintaining pregnancy (47).

The histological observation of collagen in equine placenta sections stained with PSR and observed under polarized light enabled the visualization of those fibers in the different structures. In the connective tissue, type I collagen fibers are densely grouped in bundles, playing a structural role, and promoting the connection between structures. The reticular fibers, formed predominantly by type III collagen, are finely distributed in the form of a network, playing a supporting role (33). In picrosirius red stained mare endometrium observed under a polarized light beam, fibers stained in red were identified as COL1, while fibers stained in green were considered to be COL3 (48). However, as reviewed by some other authors, this method has been proven to be uncapable to distinguish collagen types since the differences in color under the polarized light are simply related to the orientation of the collagen bundles and not to the type of collagen, rendering such interpretation obsolete (34). Polarized colors are not due to any chemical reaction from the components of Picrosirius Red stain that would enable to differentiate collagen types by the dye, but only represent fiber thickness and packing (49, 50). Therefore, since this is a particularly useful method to reveal the organization and/or heterogeneity of collagen fiber orientation in different connective tissues in normal or pathological tissues (34), this stain was used to assess collagen in mare placenta. In the present study, collagen fibers were depicted in different locations depending on the histological structure of the equine chorioallantoic. In fact, in all mares' placentas collagen fibers predominated in the chorionic plate and in the allantoic membrane, while very few were observed in the chorionic villi. The presence of collagen in the chorionic villi connective tissue was previously described in histological sections of human placenta (26). The increase in the thickness of the chorionic plate connective tissue, observed in the pregnant horn of older mares, who also had longer gestations, may explain the raise in the mRNA levels of *COL1A1, COL3A1*, and *COL5A1* and in hydroxyproline. However, further studies are needed to support this hypothesis.

We have shown that collagen deposition and microvascularization (vascular area and vessel count), increased in the gravid horn of older mares' placentas. Although in other species placenta fibrosis may indicate placental insufficiency and reduced neonate body weight, this was not observed in the present study. The increased vascularization in the gravid horn might have been a compensatory feature to provide the appropriate nutrition to the equine fetus. In the bitch, the lowest ratio of placenta weight and puppy weight occurs in larger litters and when placentas depict the highest capillary density, with respect to smaller litters (14). In older mares, the highest ratio of placenta weight and foal weight was associated with more parturitions, and placentas that presented the highest vascular density, which might show an adaptation of the placenta and increased efficiency with parity, as referred (51). Nevertheless, in aging sub-fertile mares with degenerative changes in the endometrium, placental efficiency decreases, due to the smaller area of placental exchange (18). As a matter of fact, in old mares with endometrosis, fetal chorionic villi are shorter and more irregular when compared to healthy young mares of the same gestational age (13). However, our data suggest that older fertile mares, were able to develop a functional placenta and carry on a successful pregnancy to term.

In humans, any changes in the surface area, vascularization, cell composition or thickness of the placental barrier, influence its transport capacity (52). It is known that fibrosis can affect the function of an organ by disrupting the transport of fluids and electrolytes (53). Although data obtained in this study do not support this hypothesis, it is possible that the degree of placenta fibrosis of old mares influences foal's weight at birth. Several authors have documented the appearance of degenerative changes in the equine endometrium with aging (54–56) that leads to less development of placental microcotyledons (18). A study carried out in our laboratory showed the appearance of fibrosis in the oviduct associated with endometrosis in old mares (17). Hence, it arises the hypothesis that the same could happen in the histological structure of the equine placenta. Considering the present study, as mares aged, and gestation length was prolonged, collagen fibers appeared to increase in a significant amount in the the connective tissue of the chorionic plate. It has been known that foal's weight at birth reflects the balance between fetal contact and placental efficiency (51). However, in the present work, collagen deposition in the gravid horn of older mares had a positive effect on placenta development and foals' weight, suggesting a physiological adaptation of the placenta for foal intrauterine development. This could be explained by the fact that the older mares used in this study, aged from 10 to 15 years old, were still not in the range of mares with more reproductive problems associated to endometrosis (over 15 years) and placental disfunction, as referred by others (51, 54). Furthermore, since the present study only focuses on placentas that have been obtained from successful births, placentas with histological lesions and/or dysfunctional placentas that possibly originated abortions were not considered. This may explain why there is no evident negative impact of mare's aging on foal's weight. Finally, unfortunately, it was not possible to collect the endometrial biopsies from the mares from which the placentas were collected, making it impossible to correlate the degree of endometrosis and the fibrosis present in the placenta.

In conclusion, our data suggest that older fertile mares, with more parities and longer gestation periods were able to develop a functional placenta, which was heavier, more vascularized, and with increased collagen and thickness of the chorionic plate, which enabled the delivery of heavier foals. Thus, these features might represent morphological and physiological adaptations of older fertile mares' placentas. However, research should be continued on a larger sample of equine placentas (including successful foalings and abortions), to obtain a group of young mares and another of old mares, aged quite apart from each other. It will also be extremely important to obtain the corresponding endometrial biopsies, to understand whether fibrosis in the endometrium is accompanied by fibrosis in the placenta and its pathology.

## Data Availability Statement

The original contributions presented in the study are included in the article/supplementary materials, further inquiries can be directed to the corresponding author/s.

## Author Contributions

GF-D, MR, and GA-P: conceptualization. ANS, AC, AT, JA-M, CF, MF, JF, MB, and ES: methodology. GF-D, MR, JF, MF, and GA-P: formal analysis. ANS, AC, AT, JA-M, CF, MF, and MB: investigation. ANS: writing—original draft preparation. GF-D and ANS: supervision project administration and funding acquisition. MF, MR, JF, ES, GA-P, and GF-D: writing—review and editing. All authors have read and agreed to the published version of the manuscript.

## Funding

This research was funded by the projects UIDB/00276/2020 and F-04 –CIISA MSC Internal Project.

## Conflict of Interest

The authors declare that the research was conducted in the absence of any commercial or financial relationships that could be construed as a potential conflict of interest.

## Publisher's Note

All claims expressed in this article are solely those of the authors and do not necessarily represent those of their affiliated organizations, or those of the publisher, the editors and the reviewers. Any product that may be evaluated in this article, or claim that may be made by its manufacturer, is not guaranteed or endorsed by the publisher.
